# PLoV: a comprehensive database of genetic variants leading to pregnancy loss

**DOI:** 10.1093/database/baaf037

**Published:** 2025-07-08

**Authors:** Evgeniia M Maksiutenko, Igor V Bezdvornykh, Yury A Barbitoff, Yulia A Nasykhova, Andrey S Glotov

**Affiliations:** Department of Genomic Medicine, D.O. Ott Research Institute of Obstetrics, Gynaecology, and Reproductology, Mendeleevskaya line, 3, Saint Petersburg, 199034, Russia; Department of Genomic Medicine, D.O. Ott Research Institute of Obstetrics, Gynaecology, and Reproductology, Mendeleevskaya line, 3, Saint Petersburg, 199034, Russia; Department of Genomic Medicine, D.O. Ott Research Institute of Obstetrics, Gynaecology, and Reproductology, Mendeleevskaya line, 3, Saint Petersburg, 199034, Russia; Department of Genomic Medicine, D.O. Ott Research Institute of Obstetrics, Gynaecology, and Reproductology, Mendeleevskaya line, 3, Saint Petersburg, 199034, Russia; Department of Genomic Medicine, D.O. Ott Research Institute of Obstetrics, Gynaecology, and Reproductology, Mendeleevskaya line, 3, Saint Petersburg, 199034, Russia

## Abstract

Pregnancy loss is an important reproductive health problem that affects many couples. Genetic factors play an important role in both spontaneous miscarriage and recurrent pregnancy loss, and the effect of genomic variants is recognized as one of the major causes of pregnancy loss in euploid foetuses. In this work, we extend our previous analysis of the genetic landscape of pregnancy loss and develop a Pregnancy Loss genetic Variant (PLoV) database to aggregate information about mutations that have been implicated in pregnancy loss. The database contains information about 534 genetic variants that have been observed in 421 cases across 47 studies, including foetus-only, parent-only, and trio-based studies. For each case, the database includes a detailed description of the phenotype, including ultrasound data (if provided in the original article). The genetic variants are scattered across all chromosomes in the human genome and affect a total of 292 unique genes. We provide a public access to the PLoV database at https://plovdb.ott.ru/.

**Database URL**: https://plovdb.ott.ru/

## Introduction

Human infertility is an important and frequent health issue, affecting >50–80 million couples worldwide [1]. Despite advances in diagnosis and treatment, the disease aetiology remains unexplained in >50% of cases, indicating that unknown genetic, epigenetic, and environmental factors are involved [2]. Besides inability to conceive, many couples face reproductive problems during pregnancy [3] and childbirth, including pregnancy loss (PL).

The term ‘PL’ is commonly used to cover both unintentional (spontaneous) loss and termination for foetal abnormalities. Unintentional PL includes spontaneous abortion (miscarriage), defined as foetal death prior to 20 weeks of gestation, and stillbirth, defined as foetal death at a later period of gestation [4]. Besides spontaneous PL, recurrent pregnancy loss (RPL) is an important reproductive health problem and affects 2%–5% of couples. The incidence of RPL varies between reports owing to various factors such as the differences in criteria used, as well as the structure of the population in question [5]. Back in 1976, the World Health Organization (WHO) defined the RPL as three and more consecutive miscarriages before the 22nd week of gestation or the loss of a foetus weighing <500 g [6]. Later, in 2011, in line with the WHO definition, the Royal College of Obstetricians and Gynecologists guidelines defined recurrent miscarriage as the loss of three or more consecutive pregnancies before 24 weeks of gestation, however, without imposing any limits on the foetal weight [7].

Women who have already experienced death of a foetus are at high risk for subsequent PL and recurrent foetal death, suggesting that genetics play an important role in families experiencing recurrent losses [8]. Previous studies showed that 70%–80% of sporadic spontaneous abortions were caused by an abnormal embryonic karyotype, with embryonic aneuploidies being one of the most frequent causes of miscarriage before 10 weeks of gestation [9]. Nevertheless, current standard-of-care practices do not allow the identification of the underlying cause of the condition in a large fraction of cases. Likewise, few genetic factors linked with RPL were identified. These include DNA methylation, sperm DNA fragmentation, chromosome heteromorphisms, and single nucleotide genetic variations. Nevertheless RPL is still regarded as one of the most challenging aspects in the field of reproductive medicine, owing to the fact that >50% of couples with RPL have no clear aetiological explanation [10].

In recent years, it has been suggested that genetic polymorphism may play an important role in adverse pregnancy outcomes. By now, more and more clinical studies examine DNA from products of conception, as well as the parent–offspring trio (maternal, paternal, and foetal) samples [11]. This approach has proven to be very useful to identify causal variants, clinically significant genes, and pathways that are essential for normal and abnormal pregnancy [12]. Identification of genetic variants leading to PL is extremely important both to deepen the understanding of their pathogenesis and to enable accurate prediction of the risk for each individual couple. Furthermore, accumulation of data supporting the causal role of short genetic variants in PL emphasizes the need to change standard practices of genetic testing in PL, both spontaneous and, especially, recurrent.

Successful identification of causal genetic variation using genome or exome sequencing technologies is heavily dependent on external data resources (such as those containing information about variant effect or frequency) and previous knowledge of the function of the respective genes and encoded proteins [13]. Hence, aggregation of data obtained in various studies on PL genetics is very important for efficient genetic testing in affected families. Previously, we have gathered information about the point mutations in euploid foetuses that have been reported as causal for different forms of PL [14]. This analysis has revealed important properties of PL genes, such as their broad expression pattern and a high degree of evolutionary constraint. As a way to extend this analysis, we decided to construct a curated database of genetic variation leading to PL, the Pregnancy Loss genetic Variant (PLoV) database. PLoV is an open resource that contains information from 47 studies devoted to causal variants identified in foetuses and couples experiencing PL, with a total of 534 independent genetic variants.

## Materials and methods

### Data resources

We analysed the results obtained in 47 different studies that were found in PubMed using a set of keywords: variants, pregnancy loss, recurrent miscarriage, and sequencing. We compiled a list combining all genetic variants found in these works. The dataset included 534 genetic variants observed in 421 cases: euploid foetuses at all gestation ages and couples experiencing RPL. Different methods were used for the identification of causal variants in the original studies: сhromosomal microarray analysis, whole exome and genome sequencing, molecular genetic testing with sequencing of one gene, Sanger sequencing, and screening for pathogenic single-nucleotide variations. Sequencing strategies included foetus-only sequencing, and trio- or quad-based analysis; sometimes researchers used only mother sequencing or multigeneration pedigree analysis. Additional information about the gene functions and the chromosome on which they are located was taken from the GeneCards database (https://www.genecards.org/).

### Database scheme and architecture

The PLoV database is built as a web-based application that interacts with an SQL database. Thus, the architecture of the database includes several integrated components: (i) a user interface (or ‘frontend’); (ii) the server application (or ‘backend’); and (iii) the database engine. The frontend is developed using React, SCSS, and JavaScript, with React facilitating the creation of modular and reusable user interface components, SCSS enhancing the maintainability and scalability of stylesheets, and JavaScript providing the necessary logic and interactivity for the web application. Webpack is employed as a build tool to compile JavaScript modules and other assets like SCSS into efficient bundles, thereby improving the application’s performance and manageability. On the backend (server) side of the application, the system leverages Python and Flask, a lightweight web application framework that is used to efficiently handle web requests, routing, and database interactions. The database engine is PostgreSQL 16, an open-source, object-relational database management system.

**Figure 1.**
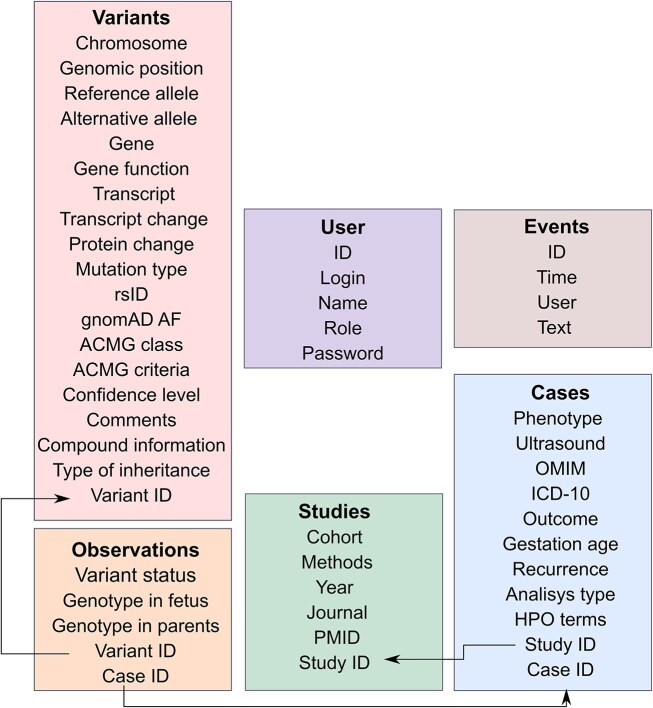
Architecture of the PLoV database. Each rectangular shape corresponds to a separate table in the database. Key fields have the ‘ID’ suffix and are connected using arrows.

The database schema includes four main tables: Variants, Cases, Studies, and Observations (Fig. 1). These tables provide detailed descriptive information about the variant and the settings and cases/families in which it was found. The Variants Table provides information about each variant (genomic location, transcript- and protein-level consequence, rsID, allele frequency according to the Genome Aggregation Database (v. 4.1 [15]), as well as information about the corresponding gene, its molecular function, and the inheritance pattern of the associated Mendelian diseases (as indicated in Online Mendelian Inheritance in Man (OMIM) or in the original study).

We also provide interpretations of variant pathogenicity according to the American College of Medical Genetics and Genomics (ACMG) criteria [16]. Variant class was determined according to the original studies, if reported by the authors. For variants with no reported ACMG class, interpretation was performed using two independent web services, Franklin (https://franklin.genoox.com/clinical-db/home) and GeneBe (https://genebe.net/about), with manual review and consideration of foetal genotype and the information on segregation in the family (if provided in the study). ACMG criteria from both automated interpretation tools and original articles were recorded. In cases when automated classification indicated stronger evidence of pathogenicity compared to the original study, variants were also manually reviewed and re-classified accordingly. Finally, each variant was manually classified into confidence classes (see the ‘Assessment of confidence levels of genetic variants’ section for details).

Each variant in the database has its own Variant ID that links the detected nucleotide substitution with the case (family) in which it was found. In the Cases Table, we provide all significant information about the foetus, parents, observed phenotypes (including Human Phenotype Ontology (HPO) terms, where available), gestational ages, and outcomes that were presented in the original articles. Each case has its own Case ID that is associated with the variants observed in this family using the Observations Table, which allows researchers to track the genotype of the parents and foetus. Each observation is assigned to a group (‘status’) according to the available information on familial segregation and genotypes (*de novo*, inherited biallelic, inherited). Variants without information on segregation were grouped according to their state in the tested family member (i.e. foetal or parental).

From the Cases section (with the help of Study ID), users can get information about specific study in which this case was described. The Studies Table contains data about the cohort and method used in the article, as well as related information that allows to find the article.

### Database availability

The user interface for working with the PLoV database has been implemented as a web application that can be freely accessed at https://plovdb.ott.ru/. A more detailed description of the user interface of the database and available options for interacting with the resource are described in the ‘Results’ section.

### Assessment of confidence levels of genetic variants

Given that some of the variants reported in the original studies had low support for their causal role in PL, we decided to manually classify each variant into three confidence categories based on the strength of supporting evidence. The criteria for inclusion into each group were as follows:

#### High-confidence variants

This group included variants with the strongest evidence of their causal role in PL. These variants were required to be classified as pathogenic or likely pathogenic and have either (i) foetal genotype information or (ii) parental genotype and gestational age information available.

#### Low-confidence variants

This group included variants that met one of the following criteria: (i) classified as benign or likely benign; (ii) classified as a variant of uncertain significance (VUS) with no foetal genotype data available; or (iii) classified as VUS with parental genotype data available, but no corresponding phenotype or gestational age information provided.

#### Moderate-confidence variants

All variants not meeting the criteria for high or low confidence were put into this category.

### Database curation and maintenance

To ensure the accuracy of the data in PLoV, users can submit genetic variant data obtained by them or suggest published articles that should be reviewed by the database curators. All submissions will be reviewed by the authors, and each entry will be marked to indicate whether it has been verified. Besides, the database will be updated every 3 months to incorporate findings from the latest research, ensuring data remain current. The authors are committed to reviewing the confidence status of variants if new pieces of evidence appear. If conflicting interpretations of pathogenicity emerge for a variant, the authors will take effort to clearly reflect this information in the database.

## Results

### Construction of the PLoV database

The PLoV database provided users with a brief description of the variants found in families who have experienced single PL or RPL, as well as support information about all cases (see the ‘Materials and methods’ section for database architecture details). To build the database, we have examined 47 published studies on PL genetics, including 36 works that employed foetal DNA for the analysis and 11 studies that only included affected couples and their family members. In total, we have collected information about 534 independent genetic variants (with different genomic position and substitution, irrespective of the number of observations) observed in 421 cases across all 47 studies.

### Descriptive statistics of the database content

**Figure 2.**
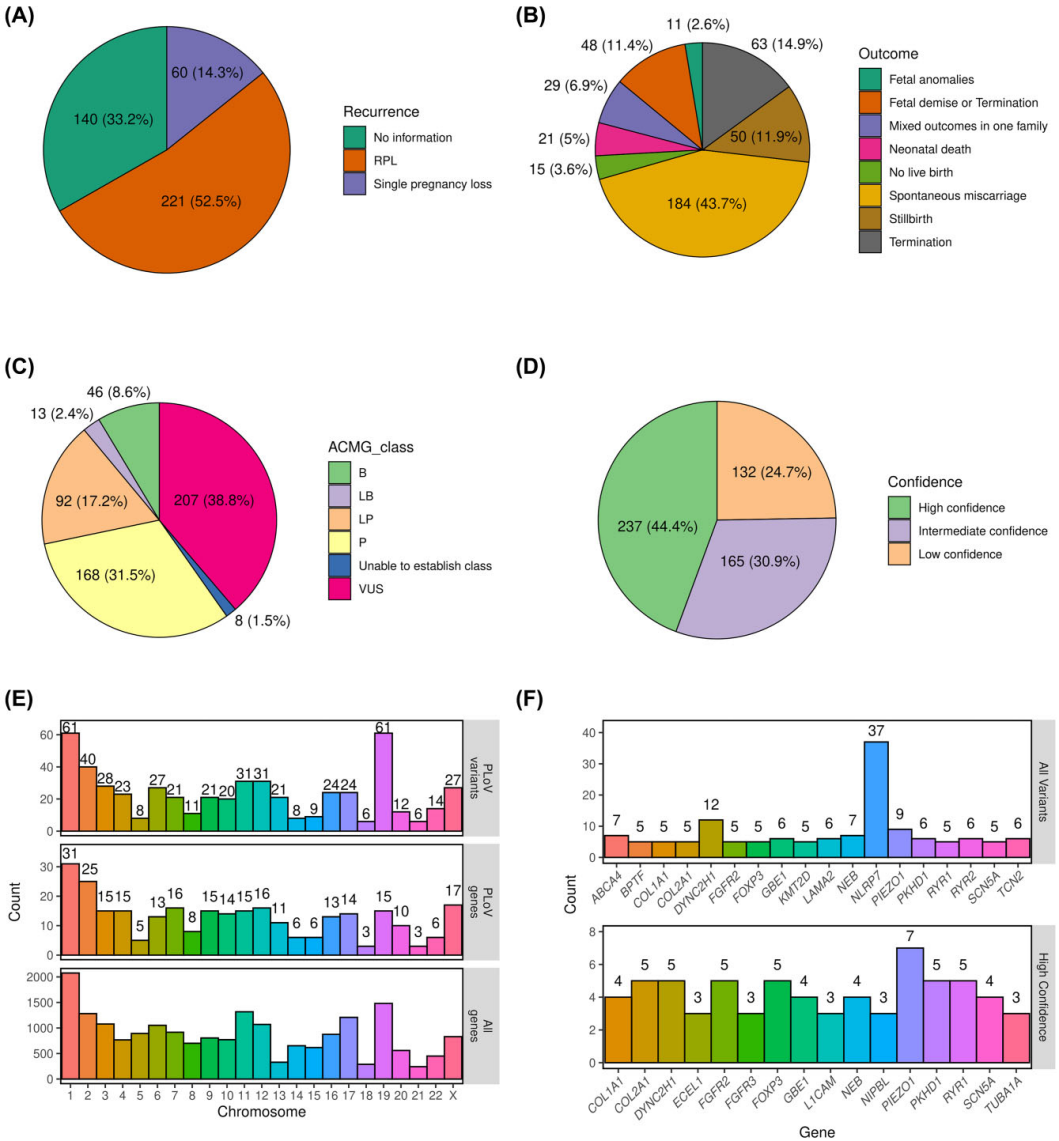
Summary statistics of the entries in the PLoV database. Pie charts showing the proportions of cases with different numbers of PLs (A) or outcomes (B). Pie charts showing the numbers and proportions of genetic variants in the indicated variant pathogenicity (C) or confidence (D) categories. (E) A bar plot showing the distribution of unique genetic variants (top) or genes (middle) across chromosomes. Total number of genes per chromosome (bottom) is included as a control. (F) A bar plot showing the number of all (top) or high-confidence (bottom) variants in the top 20 genes with the highest number of PL-causing variants.

Following the creation of the database, we next went on to summarize the proportions of different types of cases and variants included. As expected, the majority of cases (221, 52.5%) corresponded to RPL (Fig. 2A). In almost a quarter of entries, however, it was not specified whether the couple experienced RPL or a single miscarriage/stillbirth. Only a minority of cases (60, 14.3%) corresponded to clearly indicated single PL events. Among the reviewed studies, a wide range of pregnancy outcomes was described, with a single or recurrent spontaneous miscarriage occurring in almost half of cases (184, 43.7%). Other common types of PL were termination for morphological anomalies, foetal demise, and stillbirth (Fig. 2B).

Among the 534 genetic variants, pathogenic and likely pathogenic ones constituted almost half of all entries (48.7%, 260 variants in total), with VUS being the second largest group (38.8%, 207 variants) (Fig. 2C). A minor fraction of variants (59, 11%) corresponded to variants that were classified as benign or likely benign by automated variant evaluation tools; in most cases, this classification was likely due to their high frequency in the general population. In good concordance with these proportions, high-confidence causal variants (see the ‘Materials and methods’ section for details on confidence level assignment) corresponded to 44.4% of all variants (Fig. 2D). As many as 132 (24.7%) of variants, however, were classified as low confidence due to the lack of evidence for their causal role in PL.

In general, variants in PLoV were scattered on all chromosomes and the distribution of the number of variants per chromosome was in line with the chromosome lengths. The largest number of genes (Fig. 2E, top) containing causal variants was found on chromosome 1, and the smallest number observed on chromosomes 18 and 21. More than 60 variants (Fig. 2E, bottom) were localized on chromosomes 1 and 2, and <10 variants were found on chromosomes 5, 18, 21, and 22.

Among all genes present in the database, the largest number of variants (Fig. 2F, top panel) corresponded to *NLRP7*; however, all of these variants were discovered in a single study [17] devoted to mutations in this gene leading to hydatidiform mole or spontaneous abortions. None of the variants in *NLRP7*, however, were classified as high-confidence causal variants. Another top gene was the *FOXP3* gene located on the X chromosome. Eight variants were observed in *FOXP3* and, curiously, all of these were described independently in seven different articles. Variants in *FOXP3* are known to be associated with several diseases, including immunodysregulation, polyendocrinopathy, enteropathy, and hydrops fetalis [18]. When only high-confidence causal variants were considered for gene-level analysis, *PIEZO1* emerged as the gene with the greatest number of such variants (seven entries), followed by *COL2A1, DYNC2H1, FGFR2, FOXP3, PKHD1*, and *RYR1* (five entries in each gene) (Fig. 2F, bottom panel).

Given that the database includes variants that have been identified in different studies involving various analysis strategies (i.e. analysis of the foetal genome, trio-based analysis, or parent-only analysis), we next set off to evaluate the concordance of sets of genes and genetic variants identified in these studies. We specifically focused on comparing the studies that involved the foetal genome analysis with those that involved only couples (i.e. both affected or unaffected adults who have experienced PL previously). The analysis demonstrated that the genes reported in these studies have a tiny overlap, ~3% (10 genes) of all affected genes (Supplementary Fig. S1). One of the most notable genes identified as causal in both foetal genome-based and parent genome-based studies was *FGFR2*, encoding a fibroblast growth factor (FGF) receptor. This result corroborates our previous observations regarding an important role of FGF signalling in PL [14].

### Description of the PLoV user interface capabilities

We developed a web application to allow the users to browse, search, download, and upload data to PLoV. The database can be accessed via https://plov.ott.ru/. The resource is freely available for browsing without registration, and registered users can (depending on their permissions) submit their data, review and approve submissions, and edit existing database entries.

**Figure 3.**
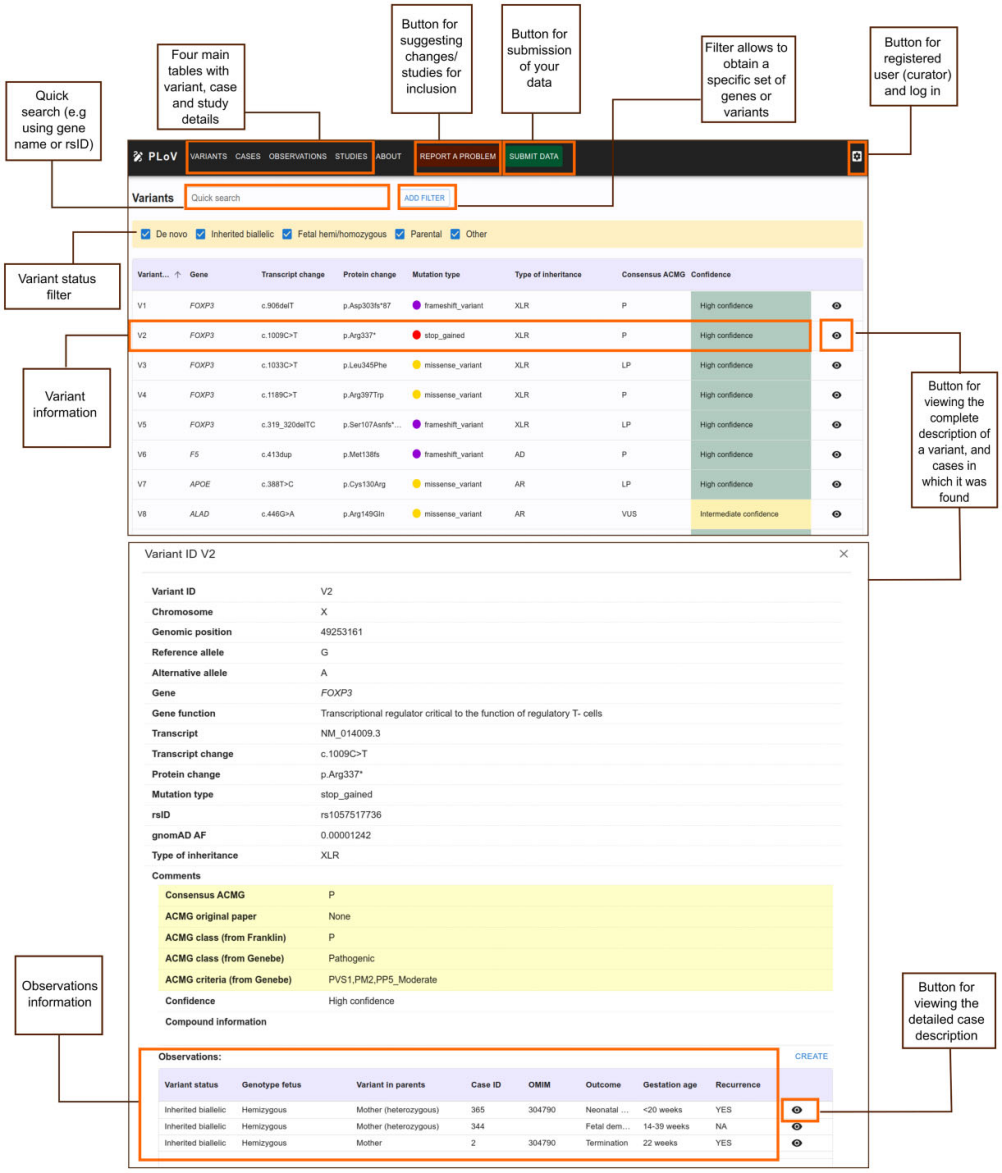
Main elements of the user interface of the PLoV database. The top screenshot shows the Variants page and functionality of the database (location of extended information panels, filtration, and data submission options). The bottom screenshot shows the detailed variant view with corresponding information about observations in different cases.

The user interface of the database consists of the main page, showing the general information about the database and the main statistics, and the three main content pages (corresponding to studies, cases, and genetic variants) (Fig. 3). Each page consists of a table showing all entries of a given type with the basic information. Extended information about each study, case, or genetic variant can be viewed in a pop-up panel, and can be edited by users with editor access (database administrators or experts). For each case, the extended information panel shows the identified genetic variants (and vice versa).

Users can submit their data to PLoV by uploading tab-separated files with entries corresponding to each of the four main tables (Studies, Cases, Variants, and Observations). The templates for submission can be downloaded directly from the database website. All submissions are evaluated by the database administrators prior to becoming available to a general viewer. Besides data submission, users can report any issues with the data using the form provided in the ‘About’ tab.

## Discussion

In recent decades, genetic variants in thousands of human genes have been established as the genetic cause of Mendelian disease. The methodology for detection of causal genetic variation in rare disease is well established [19], and it is now being applied for identification of novel sequence variants in families affected by miscarriage (including foetuses, parents, and affected trios). Multiple potentially causal variants for PL have so far been reported ranging from confidently pathogenic to almost certainly benign (according to ACMG classification [16]). Accumulation of these data necessitates aggregation, sharing, cross-assessment, and validation of detected genetic variants, especially considering the importance of the subject.

Previously, we have performed a systematic review of the genetic variants that have been linked to PL in the studies involving foetal genetic material [14]. In this work, we expand this study by setting up a PLoV database that encompasses not only studies involving the foetal genome, but also a range of works that were solely focused on studying couples affected by RPL. As shown in Supplementary Fig. S1, the sets of genes investigated in studies involving foetal DNA or only parent DNA overlap, but include a significant number of distinct genes. This result suggests that the inclusion of parent-only studies expands the set of candidate genes that should be considered when conducting molecular diagnostics in PL and RPL cases. It has to be noted that the variants reported in parent-only studies tend to be enriched with benign and likely benign variants (interpreted according to the ACMG criteria), which may indicate lesser quality of these findings. As a way to address the varying levels of supporting evidence for genetic variants included in the database, we manually assigned a confidence level to each variant.

While general-purpose genetic variant databases, such as NCBI ClinVar [20] or Human Gene Mutation Database [21], focus on genetic variants that lead to a specific postnatal phenotype, our database aggregates variants that have been observed in PL genes and may thus be absent from ClinVar or other databases. Besides a comprehensive enumeration of variants linked to PL, PLoV provides a far richer set of fields that can be used to record the details of the case, including ultrasound results. These data allow the users to efficiently filter and subset the data to obtain a specific set of genes or variants to be investigated.

Open contribution to the PLoV database ensures the continuous expansion of the dataset, which is especially important given the aforementioned rate of accumulation of new knowledge about the genetics of miscarriage and RPL. Besides, the opportunity of the user to leave feedback on the reported variants in the database could further improve the accuracy of content. We hope that both open contribution and peer review of the content could be supported by a user-friendly interface of the database that is convenient for both researchers and clinicians. We believe that all of the aforementioned features make the PLoV database a one-stop resource for specialists working with genetic testing in PL, allowing them to obtain supporting evidence for their findings. Thus, we believe that PLoV could be introduced as a tool to aid interpretation of next generation sequencing results in affected couples and the choice of reproductive strategy for couples experiencing recurrent miscarriages. Such efforts could, in turn, increase the effectiveness of early detection of risk alleles and prevention of PL.

## Supplementary Material

baaf037_Supplemental_File

## Data Availability

The database described in this article is freely available at https://plovdb.ott.ru.
