# Supplementary material for: PLoV: a comprehensive database of genetic variants leading to pregnancy loss
Source: Database (Oxford). 2025 Jul 8;2025:baaf037. doi: 10.1093/database/baaf037 (PMC12462621; doi:10.1093/database/baaf037)
Supplement: baaf037_Supplemental_File [file baaf037_supplemental_file.pdf]

PLoV: a comprehensive database of genetic variants leading to pregnancy loss

Evgeniia M. Maksiutenko[1], Igor V. Bezdvornykh[1], Yury A. Barbitoff [1], Yulia A Nasykhova[1] and Andrey S. Glotov[1]

1 - Dpt. of Genomic Medicine, D.O. Ott Research Institute of Obstetrics, Gynaecology, and Reproductology, St. Petersburg, Russia

Supplementary Figures

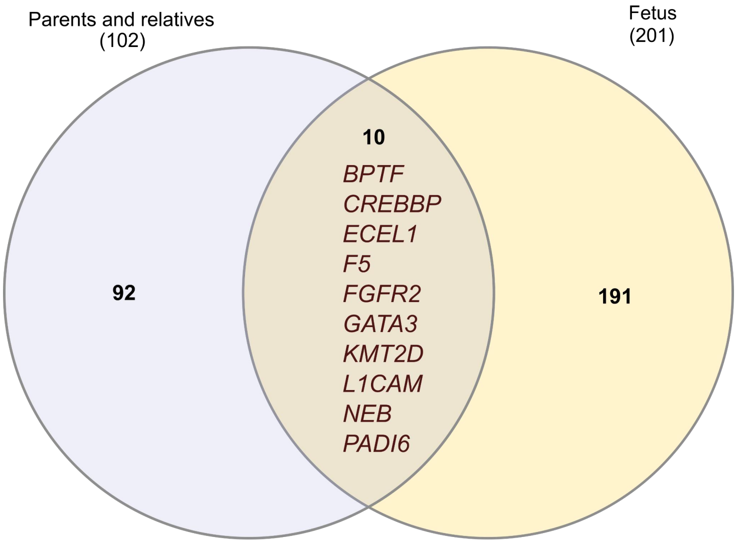

Supplementary Figure 1. A Venn diagram showing the overlap between the genes implicated in PL using fetus-only or trio-based, and parents-only approaches.
